# Prevention of pneumococcal diseases in the post-seven valent vaccine era: A European perspective

**DOI:** 10.1186/1471-2334-12-207

**Published:** 2012-09-07

**Authors:** Catherine Weil-Olivier, Mark van der Linden, Iris de Schutter, Ron Dagan, Lorenzo Mantovani

**Affiliations:** 1 Department of Pediatrics, University Denis Diderot, Paris, France; 2 Department of Medical Microbiology, National Reference Center for Streptococci, University Hospital RWTH Aachen, Aachen, Germany; 3 Department. of Pediatric Pneumology, Cystic Fibrosis Clinic and Pediatric Infectious Diseases, Universitair Ziekenhuis Brussel (UZ Brussel), Brussels, Belgium; 4 Pediatric Infectious Disease Unit, Soroka University Medical Center and Faculty of Health Sciences, Ben-Gurion University, Beer-Sheva, Israel; 5 CIRFF/Center of Pharmacoeconomics, Faculty of Pharmacy, University of Naples, Naples, Italy

**Keywords:** Pneumococcal conjugate vaccine, Invasive pneumococcal disease, Community-acquired pneumonia, Acute otitis media, Vaccine serotype coverage, Epidemiology-incidence

## Abstract

**Background:**

The burden of invasive pneumococcal disease in young children decreased dramatically following introduction of the 7-valent pneumococcal conjugate vaccine (PCV7). The epidemiology of *S. pneumoniae* now reflects infections caused by serotypes not included in PCV7. Recently introduced higher valency pneumococcal vaccines target the residual burden of invasive and non-invasive infections, including those caused by serotypes not included in PCV7. This review is based on presentations made at the European Society of Pediatric Infectious Diseases in June 2011.

**Discussion:**

Surveillance data show increased circulation of the non-PCV7 vaccine serotypes 1, 3, 6A, 6C, 7 F and 19A in countries with routine vaccination. Preliminary evidence suggests that broadened serotype coverage offered by higher valency vaccines may be having an effect on invasive disease caused by some of those serotypes, including 19A, 7 F and 6C. Aetiology of community acquired pneumonia remains a difficult clinical diagnosis. However, recent reports indicate that pneumococcal vaccination has reduced hospitalisations of children for vaccine serotype pneumonia. Variations in serotype circulation and occurrence of complicated and non-complicated pneumonia caused by non-PCV7 serotypes highlight the potential of higher valency vaccines to decrease the remaining burden. PCVs reduce nasopharyngeal carriage and acute otitis media (AOM) caused by vaccine serotypes. Recent investigations of the interaction between *S. pneumoniae* and non-typeable *H. influenzae* suggest that considerable reduction in severe, complicated AOM infections may be achieved by prevention of early pneumococcal carriage and AOM infections. Extension of the vaccine serotype spectrum beyond PCV7 may provide additional benefit in preventing the evolution of AOM. The direct and indirect costs associated with pneumococcal disease are high, thus herd protection and infections caused by non-vaccine serotypes both have strong effects on the cost effectiveness of pneumococcal vaccination. Recent evaluations highlight the public health significance of indirect benefits, prevention of pneumonia and AOM and coverage of non-PCV7 serotypes by higher valency vaccines.

**Summary:**

Routine vaccination has greatly reduced the burden of pneumococcal diseases in children. The pneumococcal serotypes present in the 7-valent vaccine have greatly diminished among disease isolates. The prevalence of some non-vaccine serotypes (e.g. 1, 7 F and 19A) has increased. Pneumococcal vaccines with broadened serotype coverage are likely to continue decreasing the burden of invasive disease, and community acquired pneumonia in children. Further reductions in pneumococcal carriage and increased prevention of early AOM infections may prevent the evolution of severe, complicated AOM. Evaluation of the public health benefits of pneumococcal conjugate vaccines should include consideration of non-invasive pneumococcal infections, indirect effects of vaccination and broadened serotype coverage.

## Background

The human nasopharynx is the reservoir of *Streptococcus pneumoniae*, which is usually carried asymptomatically, and is transmitted to other individuals by respiratory droplets. The carriage rate is highest in young children, who most likely carry pneumococci in the nasopharynx at least one time, and are the primary source for its spread within a community. In the host, pneumococci can spread locally from the nasopharynx to cause otitis media or sinusitis, or to the lungs to cause pneumonia. Pneumococci can also cause invasive infections with high mortality. Pneumonia with empyema or bacteraemia, febrile bacteraemia and meningitis are the most common invasive pneumococcal diseases (IPD). In Europe and the US, the risk of infection is greatest in children younger than 2 years, far from negligible in children under 5 years, declines steadily through the teens and then increases again after the age of 50 years [1-4].

The burden of invasive pneumococcal disease in young children decreased dramatically following the introduction of the 7-valent pneumococcal conjugate vaccine (PCV7) into national immunization programs (NIPs) in the US and a number of EU countries [5,6]. PCV7 contains capsular polysaccharides from seven *S. pneumoniae* serotypes conjugated to CRM_197_, a non-toxic mutant of diphtheria toxin. When PCV7 was licensed, those serotypes (4, 6B, 9 V, 14, 18C, 19 F and 23 F), caused the majority of invasive pneumococcal infections in the US [7] and were also associated with antibiotic resistance [8,9].

In countries with high vaccination coverage, the epidemiology of *S. pneumoniae* now reflects infections caused by a limited number of serotypes that were not included in the 7-valent vaccine. This has partially offset decreases in vaccine serotype IPD incidence. The global benefit of PCV7 programs [8,10,11] has not been affected because the increases in incidence of non-vaccine serotype infections are small compared with the decreases in incidence of IPD caused by vaccine serotypes [12,13]. Recently introduced higher-valency PCV formulations (Table 1) are intended to target the residual burden of IPD, non-invasive infections (pneumonia and otitis media, especially complicated cases) and infections caused by non-PCV7 serotypes in children up to 5 years of age. 

**Table 1:** Pneumococcal serotypes included in the licensed pneumococcal conjugate vaccines

**Vaccine**	**Manufacturer**	**Serotypes**	**Indicated age**
PCV7 (Prevenar)	Pfizer	4, 6B, 9V, 14, 18C,19F, 23F	< 5 years
PCV10 (Synflorix)	GSK	4, 6B, 9V, 14, 18C,19F, 23F, **1**, **5**, **7F**,	< 5 years
PCV13 (Prevenar 13)	Pfizer	4, 6B, 9V, 14, 18C,19F, 23F, **1**, **3**, **5, 6A**, **7F**, **19A**	< 5 years

This review is based on presentations made during a symposium at ESPID 2011 that provided an update of ongoing surveillance data, published clinical trials and effectiveness data evaluating conjugate pneumococcal vaccines. Our objective is to provide an overview of pneumococcal diseases including IPD, community-acquired pneumonia (CAP) and severe AOM in Europe in the post-PCV7 era.

### Impact of conjugate vaccines on pneumococcal invasive disease

#### Current epidemiology of invasive pneumococcal disease in Europe

PCV7 was included in the NIPs, or was recommended for routine vaccination, in a number of European countries, between 2006 and 2008 (Table 2). It is administered concomitant with other childhood vaccines as a 2- or a 3-dose primary series plus a booster [14]. Recent surveillance data in European countries having a vaccine uptake of 80-90% indicate that the PCV7 serotypes have nearly disappeared from IPD isolates in young children [15]. The data have also demonstrated increased circulation of the non-PCV7 serotypes 1, 3, 6A, 6C, 7 F and 19A [15]. This has spurred efforts to confirm the potential benefits of the broadened serotype coverage offered by recently licensed higher valency PCV vaccines. 

**Table 2:** PCV7 adoption in national immunization programs (NIP) or universal vaccination recommended by health authorities in European countries

**Country**	**Introduction in NIP or universal recommendation**	**Schedule**
Belgium	Jan 2007	2 + 1
Cyprus	Sep 2008	3 + 1
Denmark	Universal recommendation Oct 2007	2 + 1
France	Universal recommendation May 2006^1^	2 + 1 Oct 2008
Germany	Universal recommendation Jul 2006	3 + 1
Greece	Jan 2006	3 + 1
Hungary	Universal recommendation Oct 2008	2 + 1
Ireland	Sep 2008	2 + 1
Italy	Dec 2006^2^	2 + 1
Luxembourg	Feb 2003	3 + 1
Netherlands	Jun 2006	3 + 1
Norway	Jul 2006	2 + 1
Spain (Madrid)^3^	Jan 2007	3 + 1
Switzerland	Jul 2006	2 + 1
Turkey	Jan 2009	3 + 1
UK	Sep 2006	2 + 1

^1^ Limited recommendation in 2003 with 3 + 1 administration schedule.

^2.^ PCV7 was recommended regionally prior to the date of inclusion in the NIP.

^3.^PCV vaccination recommendation is regional.

In the UK, PCV7 became part of the NIP in September 2006. A recently published review reported that cumulative weekly reported cases of PCV7 vaccine-serotype IPD in children <5 years of age fell from 400 in 2006 to 25 in 2010. Cases caused by non-vaccine serotypes increased from 150 to 375 over the same period, for an overall reduction in IPD of 150 cases in 2010 compared to 2006 [13]. Reported non-vaccine serotype IPD cases among children < 2 years of age increased between 2007 and 2010 [10,13,16]. By 2011 they had returned to the numbers reported in 2008, a decrease that followed the introduction of the PCV13 vaccine [10,13,16].

PCV7 was introduced in Germany in July 2006. In children <2 years of age, approximately 100 cases of vaccine-type IPD were reported annually between 1997 and 2007. Within 2 years after widespread PCV7 vaccination, approximately 10 cases/year of vaccine-serotype IPD were reported. Most of these IPD infections occurred in unvaccinated children or in those too young to be vaccinated (German National Reference Center for Streptococci [GNRCS]; Personal communication, M. van der Linden).

German capture-recapture data show the overall incidence of IPD in those <2 years of age fell from about 20 cases/100 000 child years between 1997 and 2003 to 10/100 000 between 2007 and 2009 [17] The mean annual incidence of PCV7 serotype IPD was 13.5/100 000 child-years between 1997 and 2003, 3.5/100 000 in 2007–08 and 2/100 000 in 2008–09. Changes in incidence for non-PCV7 serotypes were not observed in the first year after implementation of PCV7 vaccination (approximately 6.5 cases/100 000), but increases were observed for some non-PCV7 serotypes, namely 1, 7 F and 19A in the second year [17,18].

#### Serotype coverage of higher valency PCV vaccines

**Figure 1:**
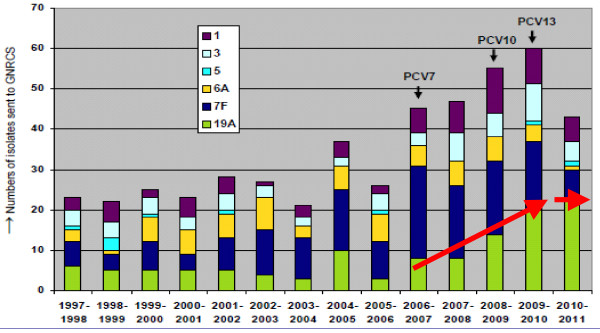
**Pneumococcal isolates of PCV13 vaccine serotypes recovered from children <2 years of age with IPD in Germany**[18]**.**

Higher valency PCV vaccines have recently been introduced in Europe, first in Germany and subsequently in Belgium, Denmark, France, Greece, Hungary, Ireland, Italy, the Netherlands, Norway, Slovakia, Sweden, the UK and Finland. They are available in Spain and Portugal, but not yet included in the NIP [14,19]. In Germany, PCV10 was introduced in April 2009 and PCV13 was introduced in December 2009. Approximately half of German children were being given PCV10 in December 2009, when PCV13 was introduced, and by 2011 about 85% were receiving PCV13 (Personal communication M. van der Linden). German IPD surveillance data are available for the non-PCV7 serotypes 1, 3, 5, 6A, 7 F and 19A from 1997 through June 2011. In 2009–2010, before introduction of PCV10, there were 107 reported cases of IPD in children <2 years of age. Of these, PCV7 serotypes were isolated from 13 cases (12%), PCV10 serotypes from 40 (37%), and PCV13 serotypes from 73 (68%). The picture was different for severe AOM in Germany at that time. Higher frequencies of serotype 3, and 19A isolates resulted in slightly increased coverage, compared to IPD, of the 13-valent vaccine relative to the 7- or 10-valent vaccines. Thus not all serotypes were represented in similar proportions in different types of pneumococcal disease. Reports of children having AOM with efflux decreased from 459 in 2008–9 to 213 in 2010–11 as the estimated vaccination rate increased from 71.9% to 84.5%. Pathogens were identified in 65 of 213 cases and *S. pneumoniae* was found in 12/65 (18.5%) [20]. Vaccine coverage of serotypes isolated from nasopharyngeal carriage was not higher than 30% for any of the three available PCV vaccines. In the UK during 2011, the cumulative weekly cases of IPD caused Xby the six new serotypes in PCV13 in children < 2 years of age fell to a lower level than before introduction of PCV7. Reports of IPD caused by any serotype not in PCV7 fell to levels similar to those in 2006 when PCV7 was introduced (Figure 1) [16]. Estimated serotype coverage of PCV7, PCV10 and PCV13 in several European countries is shown in Table 3. Recent data on the serotype distribution of IPD in European children < 2, < 5 or ≤ 15 years of age are summarized in Table 4. 

**Table 3:** Estimated vaccine serotype coverage (%) of IPD in children < 2 years of age

	**PCV7**	**PCV10**	**PCV13**
Belgium (2009)[21]	3	38	69
Netherlands (2006–2008)[22]	35	69	79
France (2008)[23]	11	34	68
Switzerland (2009)[24]	19	36	76
Portugal (2006–08)[25]	18	39	77
Germany (2009–10)[26]	12	37	68
Spain (2009–2010)[27]	4	44	80
Greece (2008–2010)[28]	12	34	73
Austria (2007–09)[29]*		62	77

* Data for Austria are in children <5 years of age.

**Table 4:** Pneumococcal serotype distribution in children < 2, < 5 or ≤ 15 years old in several European countries

**Country**	**3 most prevalent serotypes**	**Age group**
Invasive pneumococcal disease		
UK (2008–2009)[30]	7F, 19A, 1	<5 years
France (2008)[23]	19A, 1, 7F	≤15 years
Belgium (2009)[21]	1, 19A, 7F	<2 years
Portugal (2006–2008)[25]	1, 19A, 7F	≤5 years
Germany (2009–2010)[26]	7F, 1, 19A	<2 years
Greece (2008–2009)[28]	19A, 7F, 3	≤5 years
Switzerland (2009)[24]	3, 19A, 7F	<5 years
Spain (2009–2010)[27]	1, 19A, 7F	<15 years
Pneumococcal pneumonia		
Italy (2007–2009)[31]	1, 19A, 3	≤15 years
Belgium (2009)[21]	1, 3, 5	≤15 years
AOM		
Germany (2009–2010)[20]	3, 19A, 19F	Not reported
Greece (2008–2009)[28]	19A, 19F, 6A	≤5 years

**Figure 2:**
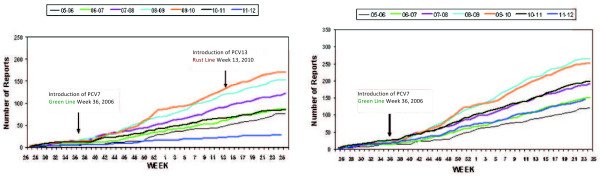
**Cumulative weekly number of reports of IPD in children < 2 years of age in England and Wales by July-June epidemiological year: 2005 to 2012.A.** IPD caused by any of the six PCV13 serotypes **B.** IPD caused by any pneumococcal serotype but not in PCV7 not in PCV7. Adapted from: Health Protection Agency, UK. Pneumococcal Disease. [16]http://www.hpa.org.uk/Topics/InfectiousDiseases/InfectionsAZ/Pneumococcal/.

Decreases in IPD cases caused by the six new vaccine serotypes contained in PCV13 were also reported in Germany by capture recapture surveillance. The number of reported IPD cases caused by serotypes 1, 3, and 7 F decreased. However, an increase in cases caused by serotype 19A that began in 2006–07 continued until 2010–11, when the rate of increase slowed. The total number of IPD cases caused by these non-PCV7 serotypes decreased from 60 in 2009–10 to just over 40 through June 2011, approximately 18 months after exclusive use of PCV13 (Figure 2) [18]. From July-December 2011 only 6 non-PCV7 serotype cases were reported (1:n = 1, 3:n = 2, 5:n = 0, 6A:n = 1, 7 F:n = 1, 19A:n = 1) [32]. It is difficult to separate the impacts of different licensed higher valency vaccines in Germany as the commercially available vaccines changed (first PCV10 only, then PCV10 and PCV13 concomitantly and finally a predominance of PCV13. Preliminary evidence, however, suggests that increased serotype coverage of higher valency vaccines is having an effect on pneumococcal infections caused by non-PCV7 serotypes, including those caused by 19A, 7 F and 6C [18,20,32,33]. 

### Pneumococcal conjugate vaccines and community acquired pneumonia

#### The burden of CAP in children

Childhood community acquired pneumonia (CAP) has significant morbidity and mortality. The burden of CAP in developed countries is 10–15 cases/1000 children per year with an annual hospital admission rate of 1–4/1000 [34]. The incidence is higher in children less than 5 years of age; 6-40/1000 compared to 7–16/1000 in children from 5–15 years of age [34]. Most deaths occur in children younger than 4 years of age [23]. Influenza, respiratory syncitial virus (RSV) and parainfluenza 1, 2 and 3 are frequent viruses causing CAP; *S. pneumoniae* is the most common bacterial cause [35].

The increasing use of pneumococcal vaccines underscores the need for accurate diagnosis in order to define the burden of pneumococcal disease and monitor vaccine effectiveness. However, the clinical differentiation of pneumonia from other lower respiratory tract infections, such as bronchitis and bronchiolitis, can be challenging because its clinical signs and symptoms, including fever, cough and tachypnea are not specific. Often children present at general practices where chest X-ray and other diagnostic methods with increased specificity are not available. The more defined the clinical endpoint, e.g., lobar pneumonia, empyema; the smaller the aetiologic diversity. Finally, there are much more published data from children hospitalized with severe or complicated CAP confirmed by X-ray and from identification of the aetiologic agent by blood culture than there are from children with less severe pneumonia.

#### Aetiology of childhood CAP

An extended discussion of methodologies used to identify causative agents of CAP is beyond the scope of this article, but it is interesting to look briefly at some studies that have compared blood culture, PCR and serology for estimating the proportion of CAP cases that are attributable to *S*. *pneumoniae.* Blood culture has some well-known limitations. It is not routinely carried out in either general practice or hospitals and has a low sensitivity of 10-30% in childhood CAP, which decreases with prior antibiotic use.

In a prospective study, PCR confirmed pneumococcal infection in 80 (10.6%) of 753 children hospitalized with a diagnosis of community acquired bacteremic pneumonia [34]. PCR allowed serotyping in blood samples from 74 of the 80 children (92.5%). More than two-thirds of those infections were caused by non-PCV7 serotypes, with serotypes 1, 3 and 19A accounting for more than half. Serotype 1 was the most frequent serotype (26/80, 32.5%) and was associated with complications (50.0% in patients with complicated pneumonia versus 18.2% in patients with uncomplicated pneumonia) and older age. Serotype 19A was second in frequency (15.0%) and was associated with younger age. Blood culture and PCR were performed simultaneously in 292 of the 753 patients. *S. pneumoniae* was identified in 47 of the 292 by one or both of the methods; in 45/292 (15.4%) by PCR but only in 11/292 (3.8%) by culture [31].

In another study, serology indicated a pneumococcal aetiology (titration of anti-pneumococcal pneumolysin and anti-pneumococcal C-polysaccharide IgG) in 18 of 101 children with CAP (18%), but blood culture was positive in only one of them [36]. In yet another study of 99 children with CAP, a pneumococcal aetiology was established in 46% by serology, in 22% by PCR and 1% by culture [37].

#### Impact of PCV7 on CAP

The impact of vaccination on CAP incidence can help define the contribution of pneumococcal vaccine serotypes to the overall disease burden. In the UK, hospital admissions for CAP among children younger than 2 years of age rose by 31%, from 1022 to 1340/million child-years between 1997 and 2006. CAP admissions then decreased by 19% to 1079/million in the two years following introduction PCV7 against a background of increasing hospital admissions of children for RTI [38].

Admissions for CAP also declined during the same period in older children who had not been vaccinated. Some of that decrease could have resulted from natural variation in the disease incidence [39]. However, herd protection likely accounted for some of the decrease, as the PCV7 vaccination coverage among those ≤2 years of age in the UK was at least 84%. Other reports have also suggested an additional impact of PCV7-vaccination on CAP incidence through herd protection [40].

In Italy, a retrospective study compared the incidence of hospital admissions in the first 2 years of life for childhood CAP in a group born in 2000–2002 before the introduction of PCV7 with a group born in 2003–2005, after its introduction [41]. For all-cause pneumonia, the incidence was 64.2/10 000 child-years among those born in 2000–2002 compared with 54.4 for those born in 2003–2005, a vaccine effectiveness of 15.2%. The corresponding results for pneumococcal pneumonia were 1.91 cases/10 000 child years versus 0.56 cases, a vaccine effectiveness of 70.5%. Only 2.9% of the all-cause pneumonia admissions in 2000–2002 were coded as pneumococcal pneumonia, but a 15.2% reduction in pneumonia by PCV7 highlights the difficulties in finding an aetiologic diagnosis of childhood CAP.

#### Empyema: complicated CAP

Empyema is an invasive complication of pneumonia. The incidence of empyema in children with CAP began to increase in both North America and in Europe in the mid to late 1990s. It has continued to increase even in those countries where PCV7 has been introduced [42-46]. Studies in Europe and the USA have consistently shown a small number of serotypes - included in PCV7 to be associated with empyema. The most frequently associated are serotypes 1, 3, 5, 7 F and 19A; which account for 50 to 85% of empyema cases, and serotype 14, accounting for up to 13% and occurring in children not vaccinated with PCV7 [47-50].

#### Estimated serotype coverage of PCVs in childhood CAP

From what is known of the pneumococcal serotypes currently associated with childhood CAP [35,51], both licensed higher valency vaccines have substantially broader serotype coverage than PCV7, i.e., more than 80% compared with 20-40%. The CAP coverage offered by PCV7 decreases from about 60% in children younger than 2 years of age to 40% in those from 2 to 5 years of age and to approximately 10% in those older than 5 years [51]. This decrease in estimated coverage did not occur for the higher valency vaccines among older children. Coverage was also estimated to be higher in complicated than in uncomplicated CAP [35]. PCV’s of higher valency thus have the potential to decrease the remaining burden of CAP and especially complicated CAP.

To summarize, CAP aetiology remains a difficult clinical diagnosis with low specificity, and wider use of diagnostic tools having increased specificity is needed. Consequently, the prevalence of CAP attributable to *S. pneumoniae* is probably underestimated. Recent reports do indicate a continuous impact of PCV7 on hospitalisations for vaccine serotype CAP. However, variations in serotype circulation and occurrence of CAP caused by non-PCV7 serotypes highlight the potential of higher valency PCVs to decrease the remaining burden of CAP.

### Conjugate pneumococcal vaccines and the aetiology of acute otitis media (AOM). An unforeseen benefit of vaccination

In addition to preventing IPD in children, PCVs reduce the carriage of vaccine serotypes in the nasopharynx, and reduce AOM caused by those serotypes. Recent investigations of the interaction between *S. pneumoniae* and non-typeable *H. influenzae* (NTHi) have increased our knowledge of the role of PCV in the prevention of AOM, particularly in complicated or difficult to treat cases.

The majority of AOM are caused by *S. pneumoniae* and NTHi [52] with NTHi infections often representing a secondary/complicated status, rather than causing a primary infection. Clinical and microbiological evaluation of over 8000 children <5 years of age showed that risks for NTHi versus *S. pneumoniae* as a single pathogen in AOM include occurrence in winter, bilaterality, >3 previous episodes and previous antibiotic treatment for AOM [53]. Mixed AOM infections or those in which NTHi plays a role are common in recurrent, non-responsive to antibiotics and chronic otitis media cases, and are more difficult to treat [54].

#### Evolution of AOM

The evolution of pneumococcal AOM is thought to begin most often with pneumococcal colonization and if local spread occurs with mucosal infection, AOM can result. The first cases of AOM are often caused by pneumococcus and are responsive to antibiotic treatment. Recurrent cases are increasingly likely to involve NTHi. Some of these cases respond to treatment, but others become chronic, with increasing likelihood of NTHi involvement [53-55]. Chronic AOM that does not eventually resolve with treatment can evolve into serious otitis media or chronic suppurative otitis media, which may result in hearing impairment or require tube insertion or surgery. The aetiology of pneumococcal AOM in over 5000 children in Israel who underwent tympanocentesis or presented with otorrhea revealed a (38%) rate of mixed pneumococcal plus NTHi infection, 59% single pneumococcal infections and 3% mixed infections with pneumococcus and other bacteria [56]. Mixed infections were associated with increasing age, previous AOM episodes, bilaterality and being a Bedouin child (possibly because of crowding in extended family households and increased carriage) [56]. This aetiology of mixed AOM is not unique to Israel, as mixed pneumococcal and NTHi infections have been observed in 19%, 29% and 40% of complicated AOM cases in studies conducted in French, Argentine and Australian aboriginal children, respectively (Cohen, personal communication, [57,58]. Determination of bacterial growth from tube otorrhea samples over an 8-day follow up of placebo or antibiotic treatment confirmed that single infection with either *H. influenzae* or *S. pneumoniae* resolved more quickly and were more responsive to antibiotics than mixed infections [59]. The patient characteristics, difficulty of treatment and survival advantages in mixed AOM are suggestive of biofilm formation, which has also been shown to play a role in other otorhinolaryngological infections [60]. In vitro and animal models have shown that NTHi can passively confer beta-lactam resistance to *S. pneumoniae* by forming biofilms or by production of beta-lactamase [61].

It is likely that NTHi is often associated with a biofilm formation in the middle ear, especially in conjunction with an initial *S. pneumoniae* infection [55,62-64]. The bacteriologic steps in AOM progression might begin with a simple *S. pneumoniae* infection at first. However, repeated, recurrent and chronic AOM could become increasingly associated with NTHi and decreasing proportions of *S. pneumoniae* and with biofilm formation.

#### The role of PCV

PCV vaccination reduces carriage of the pneumococcal serotypes that are present in the vaccine and thus prevents local spread that would otherwise cause early AOM infections [65]. If the incidence of early, simple AOM is reduced, then one would expect that there should also be a reduction in mixed and complicated AOM. In fact, looking again at an early PCV7 efficacy trial, there was relatively low overall efficacy against any AOM, but up to nearly a 23% reduction in recurrent AOM [66]. The importance of timely vaccination to prevent the first cases of AOM is highlighted by reports that while vaccination of children with a history of recurrent AOM pneumococcal vaccination reduced carriage of vaccine serotypes, it did not protect against subsequent recurrences of AOM [67-69]. A randomized trial of an investigational PCV11 vaccine has shown (by microbiological analysis of middle ear fluid) that PCV vaccination reduced the incidence of mixed pneumococcal and NTHi infection by more than 30% [70]. When comparing the pathogens recovered from middle ear fluid of PCV vaccinated versus control children with an epidemiology suggestive of complicated AOM, there were reductions of 58% in pneumococcal vaccine serotypes, 51% in non-vaccine serotypes and 35% in *H. influenzae,* which would be expected if pneumococcal vaccination reduced initial AOM infections that eventually became recurrent or chronic and mixed [70]. The PCV method included an *H. influenzae*-derived protein carrier, therefore at least some role of the NTHi antigen could not be ruled out. Two other recent observational studies showed that PCV reduced emergency room visits for AOM with mixed infection and complicated by otorrhea. They also showed that there were significant reductions in pneumococcal and NTHi isolates from middle ear fluid compared to fluid obtained prior to PCV vaccination [71]. Studies that have reported on the clinical effectiveness of PCV7 against recurrent, complicated or antibiotic-resistant AOM have recently been reviewed [72].

Preliminary results from PCV efficacy studies and post PCV epidemiology data suggest that considerable reduction of both *S. pneumoniae* and NTHi infections may be achieved by prevention of early pneumococcal carriage and AOM, decreasing the number of children with severe disease [73]. One study has already shown that PCV vaccination resulted in 40% reductions in ambulatory visits and antibiotic use for AOM [74]. Whether the presence of the *H. influenzae*-derived protein D as a carrier in PCV10 may further decrease the role of NTHi in AOM still remains to be seen. Given the evidence for serotype replacement, extension of the PCV serotype spectrum beyond PCV7 may provide additional benefit in prevention the evolution of AOM. Broadened prevention of AOM would have an important effect on cost effectiveness of vaccination [75-77].

### Public health benefits of pneumococcal vaccine

The PCV7 vaccine is licensed in over 90 countries and widely used, or included in the national immunization program (NIP), in more than 40 countries [78]. Reduction in the disease burden in vaccinated children and herd protection together with limited disease caused by non-vaccine serotypes indicated a favorable cost effectiveness ratio that influenced adoption in NIPs [79-82]. However, the cost effectiveness of PCV7 in the NIPs in some European countries may now be reduced by increases in invasive disease caused by non-vaccine serotypes [16,83-86]. Recent reviews of published PCV7 cost effectiveness evaluations highlight the contributions of herd protection, prevention of pneumonia and AOM and vaccine coverage of non-PCV7 serotypes [79,86-89]. In England and Wales, IPD surveillance data revealed that, despite increased occurrence of non-PCV7 serotype IPD (primarily caused by 7 F, 19A, and 22 F), the overall incidence of IPD in 2009–10, in those < 2 and > 65 years of age, was 56% and 19% lower, respectively, than it was before PCV7 was included in the NIP [89]. The authors concluded that further reductions should follow the use of higher valency vaccines. Most health economic models assume the cost of PCV vaccination will be recovered by future savings [79,88,90]. The direct and indirect costs associated with pneumococcal disease are high, thus vaccine serotype infections prevented by herd protection and cases caused by non-vaccine serotypes have strong effects on the cost effectiveness of pneumococcal vaccination in NIPs.

In the first 5 years of PCV7 use in the US, the vaccine was estimated to have prevented 38 000 cases of IPD at a cost of $112 000 per life-year saved [88]. When cases of IPD avoided in unvaccinated individuals were included, the total was 109 000 cases of IPD at a cost of $7500 per life year saved. A review of European data [90] identified 3 models from the UK and Norway in which analyses were performed with and without including indirect effects. Scenarios that included indirect effects i.e., herd protection yielded lower costs per life year gained (LYG) [91-93]. Considering cost per quality adjusted life year (QALY), i.e., reduced morbidity in addition to reduced mortality also consistently improved the health-economic results in favor of vaccination with PCV7 [94].

A subsequent Italian health economics analysis found PCV13 to be highly cost effective compared to no vaccination and also to PCV7 [87]. This analysis included the reduced risk of vaccinated individuals for IPD (sepsis and meningitis), pneumonia (inpatient and outpatient), meningitis, AOM (mild-moderate and severe) with or without complications or sequellae. Reductions in quality of life – essentially caused by sequelae of the diseases were considered. The model assumed vaccination coverage of 80%, clinical efficacy of 97.4% against IPD caused by PCV7 serotypes and 7% against all AOM. Assumed reductions in visit rates among children <2 years of age were 20% for AOM, 39% in cases of pneumonia and hospitalization rates and 52.4% for all-cause pneumonia [67,71,95-98]. Based on risk assessments for IPD, pneumonia, and otitis media, risks of hospital admission, complications, direct plus indirect costs of treatment, and mortality, the costs per life years saved and QALY for PCV13 were dominant compared to PCV7, primarily because it covers six additional serotypes and consequently increased projected savings by preventing infections caused by additional serotypes. In regions of Italy where PCV7 had not been introduced prior to use of PCV13 – Lombardia and Piemonte – the costs per QALY were approximately 1300 to 2100 euro, respectively. Sensitivity analysis showed that when vaccination coverage was less than 80%, or if indirect effects were excluded, the cost per QALY increased from 60 000 to 70 000 euro [87].

Pneumococcal vaccination in NIPs is a clinically- and cost-effective strategy for PCV7 and potentially more favorable for higher-valency PCVs. Coverage of at least 80% of the target population provides sufficient herd protection to gain economic benefits. Increase in invasive disease caused by non-vaccine serotypes reduces the overall direct effects of vaccination and offsets potential positive herd protection benefits in unvaccinated individuals [79,87]. The higher valency pneumococcal vaccines could have better net public health benefits than PCV-7 depending on the impact of broader serotype coverage.

## Summary

Routine PCV7 vaccination has had a major impact on the incidence of invasive and noninvasive pneumococcal diseases (IPD, CAP, AOM) in children worldwide. Here we present a European perspective at the end of the PCV7 vaccine era. PCV7 serotypes have greatly diminished among IPD isolates. The prevalence of some non-PCV7 serotypes (e.g. 1, 7 F and 19A) has increased. The introduction of pneumococcal vaccines containing a wider range of serotypes will broaden serotype coverage and is likely to have a positive and substantial impact on further decreasing the burden of CAP disease in children. Further reduction of pneumococcal carriage and early AOM infections may prevent the evolution of severe, complicated AOM. Broader serotype coverage to reduce residual disease and resulting in herd protection against non-PCV7 serotypes would increase net public health benefits.

## Competing interests

CW-O has received reimbursements or fees, from several companies including Pfizer, which supported the publication of this paper. She does not hold any stocks or shares in any company that may in any way gain or lose financially from the publication of this manuscript, and has no other financial or non-financial competing interests. MvdL has received speaker’s honoraria and research grants from, and has been a member of advisory boards for, Wyeth/Pfizer, GSK and SanofiPasteurMSD. IdS has participated in advisory boards on pneumococcal diseases and vaccines for Wyeth-Pfizer and GlaxoSmithKline. She has participated in advisory boards on NTHi for GSK, is a member of a steering committee of a research project sponsored by Wyeth-Pfizer and has been a speaker for Pfizer on pneumococcal pneumonia. RD has received grants/research support from Berna/Crucell, Wyeth/Pfizer, MSD, Protea; has been a scientific consultant for Berna/Crucell, GlaxoSmithKline, Novartis, Wyeth/Pfizer, Protea, MSD and a speaker for Berna/Crucell, GlaxoSmithKline, Wyeth/Pfizer; he is a shareholder of Protea/NASVAX”. LM has received speaker’s fees on pneumococcal diseases and vaccines for Pfizer, participated in advisory boards for GSK in areas other than vaccines and has received research grants from Pfizer in therapeutic areas other than vaccines.

## Authors’ contributions

This article is based on a series of presentations delivered during an industry-supported symposium at the European Society for Paediatric Infectious Diseases (ESPID) at den Haag the Netherlands in June, 2011. MvdL had primary responsibility for content selection, interpretation and development of the section on the impact of conjugate vaccines on pneumococcal invasive disease, IdS for pneumococcal conjugate vaccines and community acquired pneumonia, RD for conjugate pneumococcal vaccines and the aetiology of acute otitis media, LM for the public health benefits of pneumococcal vaccine. CW-O took primary responsibility for the background, provided significant guidance for discussion of the impact of conjugate vaccines on IPD, and critical evaluation and revision of the entire manuscript. All authors contributed to revision, and approved the final manuscript.

## Pre-publication history

The pre-publication history for this paper can be accessed here:

http://www.biomedcentral.com/1471-2334/12/207/prepub
